# Genome-Wide Association Study of Egg-Laying Traits and Egg Quality in LingKun Chickens

**DOI:** 10.3389/fvets.2022.877739

**Published:** 2022-06-20

**Authors:** Jinfeng Gao, Wenwu Xu, Tao Zeng, Yong Tian, Chunqin Wu, Suzhen Liu, Yan Zhao, Shuhe Zhou, Xinqin Lin, Hongguo Cao, Lizhi Lu

**Affiliations:** ^1^State Key Laboratory for Managing Biotic and Chemical Threats to the Quality and Safety of Agro-Products, Institute of Animal Science and Veterinary, Zhejiang Academy of Agricultural Science, Hangzhou, China; ^2^College of Animal Science and Technology, Anhui Agricultural University, Hefei, China; ^3^Wenzhou Vocational College of Science and Technology, Wenzhou, China; ^4^Wenzhou Golden Land Agricultural Development Co., Ltd., Wenzhou, China

**Keywords:** GWAS, chicken, egg-laying, egg quality, candidate gene

## Abstract

Egg production is the most important trait of laying hens. To identify molecular markers and candidate genes associated with egg production and quality, such as body weight at first oviposition (BWF), the number of eggs produced in 500 days (EN500), egg weight (EW), egg shell thickness (EST), egg shell strength (ESS), and Haugh unit (HU), a genome-wide analysis was performed in 266 LingKun Chickens. The results showed that thirty-seven single nucleotide polymorphisms (SNPs) were associated with all traits (*p* < 9.47 × 10^−8^, Bonferroni correction). These SNPs were located in close proximity to or within the sequence of the thirteen candidate genes, such as Galanin And GMAP Prepropeptide (GAL), Centromere Protein (CENPF), Glypican 2 (GPC2), Phosphatidylethanolamine N-Methyltransferase (PEMT), Transcription Factor AP-2 Delta (TFAP2D), and Carboxypeptidase Q (CPQ) gene related to egg-laying and Solute Carrier Family 5 Member 7 (SLC5A7), Neurocalcin Delta (NCALD), Proteasome 20S Subunit Beta 2 (PSMB2), Slit Guidance Ligand 3 (SLIT3), and Tubulin Tyrosine Ligase Like 7 (TTLL7) genes related to egg quality. Interestingly, one of the genes involved in bone formation (SLIT3) was identified as a candidate gene for ESS. Our candidate genes and SNPs associated with egg-laying traits were significant for molecular breeding of egg-laying traits and egg quality in LingKun chickens.

## Introduction

It is well-known that egg-laying traits and egg quality are very important economic characteristics in the chicken industry and are greatly associated with both the breeding value and retail egg value. Currently, the release of the chicken quantitative trait locus (QTL) contains 16,271 QTLs from 367 publications, which represent 442 different traits (https://www.animalgenome.org/cgi-bin/QTLdb/GG/index). However, despite a range of studies in this area, wide confidence intervals (CIs) for the positions of QTL remain that have rarely been replicated. A new research era was initiated with advances in sequencing technology and single nucleotide polymorphism (SNP) chip, and genome-wide association study (GWAS) has become one of the most effective methods to detect genetic variation in livestock. In previous studies, two SNPs related to body weight at first oviposition (BWF) were located at 78.8 and 78.9 Mb, respectively, on the GGA4 gene by GWAS of Jinghai Yellow chicken, and both were located in FAM184B gene (1). Liu et al. (2) performed a GWAS in 1,078 Rhode Island Red hens and found that the 117.87–118.36 Mb region of the GGA1 was associated with the number of eggs produced (EN) in hens 37–72 weeks that include POLA1, PDK3, PRDX4, and APOO genes. Liu et al. (3) discovered that the GTF2A1 and CLSPN genes may be candidate genes associated with EN by influencing ovarian and uterine functions. For egg quality traits, Wolc et al. (4) reported that a 90 kb genomic region (169.42–169.51 Mb) in GGA1 is associated with egg weight (EW) at 28–66 weeks of age, two promising genes (DLEU7 and MIR15A) can be mapped to this narrow significant region and may affect EW in a pleiotropic manner. Sun et al. (5) discovered a genomic region spanning from 57.3 to 71.4 Mb in GGA1 that is significantly associated with eggshell quality and includes ITPR2, PIK3C2G, and NCAPG genes. In addition, previous studies have shown that a genomic region spanning from 8.95 to 9.31 Mb (≈0.36 Mb) on GGA13 was significantly associated with the Haugh unit (HU), Meanwhile, MSX2 and DRD1 genes were related to embryo development and egg production (6).

The LingKun chicken is a Chinese indigenous chicken breed, which has the characteristics of large body, early sexual maturity, good meat quality, disease resistance, especially excellent egg production performance, and good egg quality. However, the genetic mechanism of these dominant traits has not been well-analyzed, so the market for Lingkun chicken has not been fully tapped, just partially reared in the Wenzhou area of Zhejiang Province. However, there are no reports on GWAS research on laying traits and egg quality of Lingkun chickens. In the present study, a GWAS was performed on 266 LingKun Chickens aged 72 weeks to identify molecular markers and candidate genes affecting reproductive traits by using whole-genome resequencing. Results could potentially benefit research into chicken reproductive traits. These promising loci and genes could be helpful to engineer practical breeding programs for the improvement of egg quality for old hens to meet the need of prolonging the laying cycle.

## Results

### Egg Production and Quality of Lingkun Chickens

**Figure 1:**
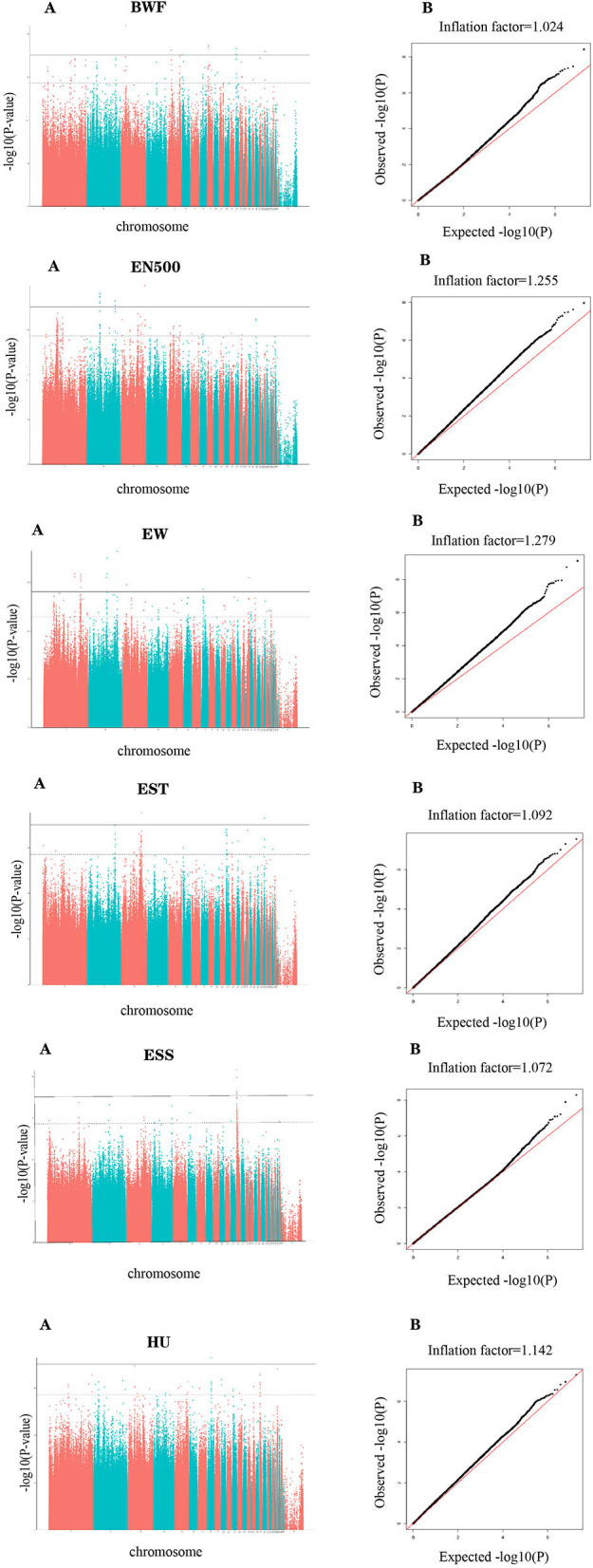
Manhattan **(A)** and Q-Q plots **(B)** were derived from GWAS for body weight at first oviposition (BWF), number of eggs produced in 500 days (EN500), Egg weight (EW), egg shell thickness (EST), egg shell strength (ESS), and Haugh unit (HU). Each dot on this figure corresponds to an SNP within the dataset, while the horizontal solid and dotted lines denote the genome-wide significance (9.47E-08) and suggestive significance thresholds (1.89E-06), respectively. The Manhattan plot contains –log10 observed *p*-values for genome-wide SNPs (y-axis) plotted against their corresponding position on each chromosome (x-axis), while the Q-Q plot contains expected –log10-transformed *p*-values. Use plotted against observed –log10 transformed *p*-values.

The basic statistics for egg-laying traits and egg quality in the Lingkun chicken for the GWAS are shown in Table 1. There are six traits that include BWF, the number of eggs produced in 500 days (EN500), EW, egg shell thickness (EST), egg shell strength (ESS), and HU. Heritability analysis showed that eggshell strength exhibited the highest heritability, with a value of 0.51, while BWF showed the lowest heritability, with a value of 0.05. EW and EN500 showed moderate heritability, with values of 0.37 and 0.38, respectively. Correlation analysis between traits is shown in Table 2, EST and EW are significantly correlated (*p* = 0.192), and ESS and EST are significantly correlated (*p* = 0.555). Meanwhile, EST and ESS were negatively correlated with EN500. Meanwhile, Manhattan diagram (A) and QQ diagram (B) of correlation analysis results are shown in Figure 1B. It showed that all the traits in this study had significant correlation SNPs, and the selection of analysis model was reasonable.

**Table 1:** Descriptive statistics for production and egg quality traits.

**Traits^**a**^**	**N^**b**^**	**Max**	**Min**	**Mean**	**SD^**c**^**	**h^**2d**^**
BWF (g)	266	1,372	1,871	1,552.68	74.47	0.05
EN500	266	111	287	197.88	34.44	0.38
EW (g)	266	40.49	70.40	52.81	5.1	0.37
EST (mm)	266	0.22	0.50	0.34	0.05	0.20
ES (Kg/cm^2^)	266	1.36	5.97	4.09	1.02	0.51
HU	266	37.85	104.37	67.28	13.55	0.43

^a^*BWF, body weight at first oviposition; EN500, the number of eggs produced in 500 days; EW, egg weight; EST, egg shell thickness; ESS, egg shell strength; HU, Haugh unit*.

*^b^Number of samples*.

*^c^Standard deviation (SD)*.

*^d^Heritability estimated*.

**Table 2:** Trait correlation analysis.

**Traits**	**BWF**	**EN500**	**EW**	**EST**	**ESS**	**HU**
BWF	1					
EN500	−0.037	1				
EW	−0.032	0.015	1			
EST	0.008	−0.101	0.192**	1		
ESS	0.062	−0.086	0.061	0.555**	1	
HU	−0.066	0.003	0.003	0.023	−0.028	1

^**^Was significantly correlated at 0.01 level (bilateral).

*BWF, body weight at first oviposition; EN500, the number of eggs produced in 500 days; EW, egg weight; EST, egg shell thickness; ESS, egg shell strength; HU, Haugh unit*.

### Body Weight at First Oviposition

A total of eight significant loci distributed on chromosomes 3, 5, 6, 9, 14, and 23 were associated with BWF (Figure 1A; Table 3). The most associated SNP G21147253A (*p*-value = 3.84E-09) was located on chromosome 3 at position 21,147,253 bp, which was found within the Centromere Protein (*CENPF*) gene, an associated SNP G16754641A (*p*-value = 5.74E-08) located on chromosome 5 at position 16,754,641 bp was found to be within the upstream of the Galanin And GMAP Prepropeptide (*GAL*) gene, two significant SNPs, G3321921T (*p*-value = 3.42E-08) and G3321930A (*p*-value = 4.42E-08), were located at 3,321,921 and 3,321,921 bp on chromosome 9, respectively, and both were within the Glypican 2 (*GPC2*) gene, and two significant SNPs, T4287370A (*p*-value = 4.84E-08) and C5246144G (*p*-value = 9.00E-08), were located at 4,287,370 and 5,246,144 bp on chromosome 14, respectively, which were found to be within the upstream of the Cytochrome P450 (*CYP3A4*; Family 3, Subfamily A, Member 4) and Phosphatidylethanolamine N-Methyltransferase (*PEMT*) gene. An associated SNP G2854459T (*p*-value = 6.53E-08) was located on chromosome 23 at position 2,854,459 bp, which was found to be within the downstream of the Transmembrane Protein 200B (*TMEM200B*) gene.

**Table 3:** Statistics for significant SNPs and proximal genes.

**Traits**	**SNP ID^**a**^**	**Chr^**b**^**	**Pos (bp)**	**Alleles^**c**^**	**MAF^**d**^**	***P*-value**	**proximal genes**
BWF	G21147253A	3	21,147,253	G/A	0.10	3.84E-09	CENPF
	G16754641A	5	16,754,641	G/A	0.11	5.74E-08	8kb U^d^ GAL
	G623515A	6	623,515	G/A	0.16	9.30E-08	—
	C3321921T	9	3,321,921	C/T	0.29	3.42E-08	GPC2
	G3321930A	9	3,321,930	G/A	0.30	4.24E-08	GPC2
	T4287370A	14	4,287,370	T/A	0.13	4.84E-08	0.7kb U CYP3A4
	C5246144G	14	5,246,144	C/G	0.27	9.00E-08	18kb U PEMT
	G2854459T	23	2,854,459	G/T	0.08	6.53E-08	30kb D^e^TMEM200B
EN500	T57212637A	2	57,212,637	T/A	0.11	3.29E-08	—
	A57212781G	2	57,212,781	A/G	0.11	6.55E-08	—
	A57924869T	2	57,924,869	A/T	0.12	3.50E-08	DCDC2
	A57925378C	2	57,925,378	A/C	0.12	2.41E-08	DCDC2
	A57945258T	2	57,945,258	A/T	0.06	5.61E-08	NRSN1
	C126998745A	2	126,998,745	C/A	0.14	8.37E-08	CPQ
	T126998774C	2	126,998,774	T/C	0.14	5.19E-08	CPQ
	C108380147G	3	108,380,147	C/G	0.19	1.08E-08	62kb D TFAP2D
EW	C137349071T	1	137,349,071	C/T	0.15	1.63E-08	SLC5A7
	T137349076C	1	137,349,076	T/C	0.16	1.15E-08	SLC5A7
	T165331309C	1	165,331,309	T/C	0.11	2.72E-08	—
	C165333250A	1	165,333,250	C/A	0.13	2.02E-08	—
	A165336526C	1	165,336,526	A/C	0.11	1.26E-08	—
	T165366719C	1	165,366,719	T/C	0.11	1.62E-08	—
	T165434400A	1	165,434,400	T/A	0.09	5.50E-08	—
	A79958113G	2	79,958,113	A/G	0.13	1.73E-08	8kb D UPP1
	G82974373A	2	82,974,373	G/A	0.09	1.78E-09	—
	C83033381T	2	83,033,381	C/T	0.09	1.13E-08	46kb U FHOD3
	A128890264G	2	128,890,264	A/G	0.14	7.55E-10	60kb DNCALD
	C17452066T	3	17,452,066	C/T	0.38	4.24E-08	ENAH
	T6405164C	8	6,405,164	T/C	0.13	7.24E-08	CEP350
	C8498988T	17	8,498,988	C/T	0.17	1.78E-08	69kb D RNF2
ESS	A5358928G	13	5,358,928	A/G	0.27	7.99E-08	SLIT3
	A5360528G	13	5,360,528	A/G	0.30	8.18E-08	SLIT3
	T5362672G	13	5,362,672	T/G	0.27	1.28E-08	SLIT3
	G5362697C	13	5,362,697	G/C	0.28	6.16E-08	SLIT3
EST	G89374390A	3	89,374,390	G/A	0.47	2.81E-08	—
	C4415750T	23	4,415,750	C/T	0.20	5.04E-08	2kb U PSMB2
HU	A17272906G	13	17,272,906	A/G	0.35	5.09E-08	90kb D TTLL7

^a^*Allele polymorphism and position on chromosome were used as SNP identity*.

*^b^The positions are based on WASHUC2*.

*^c^The first allele is minor allele*.

*^d^Gene frequency of the minor allele*.

*^e^U, upstream of; D, downstream of*.

*BWF, body weight at first oviposition; EN500, the number of eggs produced in 500 days; EW, egg weight; EST, egg shell thickness; ESS, egg shell strength; HU, Haugh unit; MAF, minor allele frequencies*.

### The Number of Eggs Produced in 500 Days

A total of seven significant loci distributed on chromosomes 2 and 23 were associated with *EN500*. The most associated SNP C108380147G (*p*-value = 1.08E-08) was located on chromosome 3 at position 108,380,147 bp was found 62 kb downstream of the Transcription Factor AP-2 Delta (*TFAP2D*) gene. Seven significant SNPs were located within the 69.78 Mb segment (between 57.21 and 126.99 Mb) on chromosome 2. In between, two significant SNPs, A57924869T (*p*-value = 3.50E-08) and A57925378C (*p*-value = 2.41E-08), were located at 57,924,869 and 57,925,378 bp, respectively, and both were within the *GPC2* gene, an associated SNP A57945258T (*p*-value = 5.61E-08) was located at position 57,945,258 bp, which was found within the Neurensin 1 (*NRSN1*) gene, two significant SNPs, C126998745A (*p*-value = 8.37E-08) and T126998774C (*p*-value = 5.19E-08) were located at 12,699,8745 and 126,998,774 bp and both were within the Carboxypeptidase Q (*CPQ*) gene.

### Egg Weight

A total of fourteen significant loci distributed on chromosomes 1, 2, 3, 8, and 17 were associated with EW. Seven significant SNPs were located within 28.08 Mb segment (between 137.34 and 165.43 Mb) on chromosome 1, in between, two associated SNPs, C137349071T (*p*-value = 1.63E-08) and T137349076C (*p*-value = 1.15E-08), located at positions 137,349,071 and 137,349,076 bp, were found to be within the Solute Carrier Family 5 Member 7 (*SLC5A7*) gene. Four significant SNPs were located within 48.93 Mb segment (between 79.95 and 128.89 Mb) on chromosome 2. In between, 79958113G (*p*-value = 1.15E-08), C83033381T (*p*-value = 1.15E-08), and 128890264G (*p*-value = 1.15E-08) were located at 79,958,113, 83,033,381, and 128,890,264 bp, respectively, which were found to be within the downstream of Uridine Phosphorylase 1 (*UPP1*) gene, Formin Homology 2 Domain Containing 3 (FHOD3) gene, and Neurocalcin Delta (*NCALD*) gene. The associated SNP C17452066T (*p*-value = 4.24E-08) was located on chromosome 3 at position 17,452,066 bp, which was found within the Actin Regulator (*ENAH*) gene, an associated SNP T6405164C (*p*-value = 7.24E-08) located on chromosome 8 at position 6,405,164 bp was found to be within the Centrosomal Protein 350 (*CEP350*) gene, In addition, an associated SNP C8498988T (*p*-value = 1.78E-08) was located on chromosome 17 at position 8,498,988 bp, which was found within the Ring Finger Protein 2 (*RNF2*) gene.

### EST and ESS

Two significant SNPs were associated with EST, the most associated SNP C4415750T (*p*-value = 2.81E-08) was located on chromosome 23 at position 4,415,750 bp, which was found within the upstream of the Proteasome 20S Subunit Beta 2 (*PSMB2*) gene. A total of four SNPs (A5358928G, A5360528G, T5362672G, and G5362697C) were significantly associated with ESS, which were located within the 3,769 kb segment (between 5,358,928 and 5,362,697 bp) on chromosome 13, which were found within the Slit Guidance Ligand 3 (*SLIT3*) gene.

### Haugh Unit

The associated SNP A17272906G (*p*-value = 5.09E-08) was located on chromosome 13 at position 17,272,906 bp, which was found within the downstream of Tubulin Tyrosine Ligase Like 7 (*TTLL7*) gene.

## Discussion

There has been an effective use of GWAS in egg-laying traits and egg quality traits in other species and narrow regions or SNPs associated with duck production trait have been revealed (7, 8). Here, we present a GWAS of egg-laying traits and egg quality in a LingKun chicken population from China. A total of 37 significant SNP loci were detected in this study. Although EW, ESS, ESS, and EST traits were significantly correlated, GWAS results did not find a common QTL.

### Body Weight at First Oviposition

The BWF reflects the constitution of the reserved chicken, the greater the BWF, the greater the energy reserve of the chicken, the greater the EW, and the stronger the ability to continue laying eggs during the laying period. Hens begin to lay eggs after sexual maturity and are still in the development stage, and their weight can still increase by 30–40 g per week. After 20 weeks, about 40 weeks of age, the growth and hair breeding were basically stopped. Xie et al. (9) performed a GWAS with F2 chickens and found that the region 169–179 Mb on GGA1 was significantly related to 23 growth traits. Jin et al. (10) found that 18 SNPs reached 5% Bonferroni genome-wide significance with growth traits in Yancheng chickens, and these SNPs, which were located on four different chromosomes and in a region of 72.3–82.1 Mb on GGA4, had a significant effect on growth traits. In the present study, growth-related genes, GAL, CENPF, and GPC2, obesity-related gene, PEMT, and CYP3A4, P450, TMEM200B genes were found to be associated with BWF. Growth and development are affected by the regulation level of hormones. The GAL gene encodes a neuroendocrine peptide, small neuropeptides that regulate a variety of physiological functions, the release of growth hormone and insulin, and adrenal gland secretion (11). CENPF has a potential role in regulating cell differentiation in skeletal myogenesis and embryogenesis (12–14). CENPF-depleted cells show delayed mitosis and reduced stability of centromeric microtubules (14, 15). It is well-known that changes in body weight are related to the mitotic differentiation of cells. GPC2 belongs to the GPC (GPC1-6) family, which plays a variety of roles in growth factor signaling and cancer cell growth (16, 17), regulates the growth and development process of the body. PEMT gene activity involves a number of physiological processes, such as lipid flow between the liver and plasma and the delivery of essential fatty acids to the blood and peripheral tissues through liver-derived lipoproteins. The PEMT gene encodes an enzyme that converts phosphatidylethanolamine to phosphatidylcholine through sequential methylation in the liver. PEMT deficiency reduces choline availability (18). In studies on childhood obesity genes, the PEMT gene was found to be associated with polyunsaturated fatty acids in human erythrocytes (19). In conclusion, *de novo* synthesis of choline through PEMT plays an important role in regulating systemic energy metabolism, and the PEMT gene may be a pharmacological target for the treatment of obesity and insulin resistance. We believe that PEMT has an indirect effect on BWF by influencing choline regulation. In addition, the CYP3A4 gene encodes a member of the cytochrome P450 superfamily of enzymes. The cytochrome P450 proteins are monooxygenases that catalyze many reactions involved in drug metabolism and synthesis of cholesterol, steroids, and other lipids. TMEM200B is a transmembrane protein overexpressed in the endometrium.

### The Number of Eggs Produced in 500 Days

Regions associated with numbers of eggs produced or egg production rate have been detected on many chromosomes, while most of these regions were population specific, only the regions on GGA2, 4, and 5 were detected in multiple studies (20, 21). Yuan et al. (3) detected nine genome-wide loci that significantly affected egg number during the laying phases of 21–26, 27–36, and 37–72 weeks using GWAS technology. Liao et al. (22) found a genome-wide significant locus (ss1985401199) located on the sex chromosome Z, which was associated with egg number in hens at 25–45 weeks. In the present study, four proximal genes (TFAP2D, DCDC2, NRSN1, and CPQ) were found, although most have not been previously reported in chicken. TFAP2D gene is the most diverse member of AP-2 family and plays a key role in development, such as the development of eyes, face, limbs, and neural tube, and is mainly expressed in the retina and brain during chicken embryo development (23). The 69.78 Mb (between 57.21 and 126.99) region on GGA2, which was considered as a QTL, was significantly correlated, which includes seven associated SNP loci, DCDC2 gene polymorphism or intron deletion may be involved in neural development and may affect signal transduction of primary cilia (24, 25). NRSN1 gene is related to chicken brain synaptic development, which plays an important role in nerve organelle transport, nerve signal transduction, or nerve growth. Since the function of these genes in chickens is unclear, we infer they may regulate egg production by interacting with the central nervous system. In addition, the CPQ gene, which is also known as plasma glutamate carboxypeptidase (PGCP), is a secreted protein hydrolyzing the circulating peptides in the extracellular microenvironment (26) that liberates I-thyroxine (T4) from thyroglobulin (Tg) and is converted to 3,5,3′-triiodothyronine (T3) by type I 5′-deiodinase (DIO1) (27). The thyroid hormone T3 binds to thyroid hormone receptors (TRs), and after heterodimerization with retinoid, X receptor can act as a transcription factor mediating diverse physiological processes, such as embryonic development, cell differentiation, metabolism, and cell proliferation, by regulating the expression of the downstream target genes (28). The regulation of thyroid and gonad is realized through hypothalamus pituitary thyroid/ovarian axis. Therefore, it is suggested that the CPQ gene may affect egg production by regulating hormone levels.

### Egg Weight

Egg weight displays a consecutive increase with the hen's age. It is known that the starting weight will affect the EW. Chickens that are fed a high-fat diet have large body weight, high liver fat content, fallopian tube weight, and EW. Liao et al. (22) discovered an SNP (ss1985401190) located on GGA4, which was significantly associated with EW. Liu et al. (4) found through GWAS that DLEU7 and MIR15A genes had a great influence on EW. In the present study, seven genes (SLC5A7, NCALD, UPP1, FHOD3, ENAH, CEP350, and RNF2) containing near SNPs are associated with EW. The biological process of the SLC5A7 gene includes affecting the development of uterine embryos. Its pathway includes the transport of glucose and other sugars, bile salts and organic acids, metal ions, and amine compounds mediated by transmembrane transport. These substances participate in metabolism and affect EW by affecting the formation of egg yolk. Meanwhile, the SLC5A7 gene responds to the depolarization of the cholinergic terminal by increasing the protein density in the synaptic constitution membrane, and choline is transported from the cell to the synaptic terminal to synthesize acetylcholine, which can mediate the synthesis of acetylcholine in cholinergic neurons. Sato et al. (29) showed that osteoblasts express specific acetylcholine receptors and cholinergic components and that acetylcholine plays a possible role in regulating the proliferation and differentiation of osteoblasts. In conclusion, the SLC5A7 gene can regulate the growth and development of hens and affect EW. NCALD is involved in the calcium signal pathway and G protein-coupled receptor signal pathway. Experiments have shown that NCALD is a potential hippocampal memory-related factor related to obesity (30). Total fat intake has an important impact on the low-density lipoprotein (LDL) peak particle diameter (LDL-PPD); LDL will lead to the increase of lipid levels in hens. When hyperlipidemia is high, it will cause insufficient blood supply to the placenta, thus affecting the laying weight. Rudkowska et al. (31) showed that NCALD with dietary fat intake influences the variation in the LDL-PPD. UPP1 gene is a dimeric enzyme and plays an essential role in pyrimidine salvage and regulation of uridine homeostasis. FHOD3 is a sarcomeric protein highly expressed in the cardiac tissue and required for the assembly of the contractile apparatus. Members of the ENAH gene family are involved in actin movement involved in a range of processes dependent on cytoskeleton remodeling and cell polarity. ENAH can activate mitogen-activated protein kinase (MAPK) and activate the p38-mapk signaling pathway. The product of the CEP350 gene is a large protein with a cytoskeleton-associated protein-glycine-rich domain (Cap-Gly), usually found in cytoskeleton-associated proteins. CEP350 gene was found to be expressed before oocyte maturation and disappeared after maturation. It is suspected to be related to the follicular formation (32). The protein encoded by the RNF2 gene is one of the Protein Crystal Growth (PcG) proteins, which is important for the transcriptional inhibition of various genes involved in the development and cell proliferation. Studies in mice have shown that the gene is involved in cell proliferation during early embryonic development. They regulate various developmental processes by promoting cell proliferation, inhibiting differentiation, and blocking cell fate regulation through many signaling pathways. It is speculated that these genes may affect EW during embryogenesis and organogenesis.

### EST and ESS

Eggshell quality is an important index to reflect the damage resistance rate of eggs and is related to egg freshness and hatching rate. During the process of eggshell formation, uncalcified eggs are bathed in uterine fluid that plays regulatory role in eggshell calcification. The difference in protein abundance plays essential roles in influencing eggshell strength. Sun et al. (33) found that 15 proteins mainly related to eggshell matrix-specific proteins, calcium binding and transportation, protein folding and sorting, bone development or diseases, and thyroid hormone activity were considered to have a closer association with the formation of strong eggshell. A lots of QTL regions affecting eggshell quality were discovered by previous linkage studies and mainly distributed on GGA 1–9 and GGA11 (32). In the present study, the promising genomic region had no overlap with the previously reported regions; PSMB2 is a candidate gene for EST revealed by the present GWA analysis. PSMB2 is one of the fourteen 20 S proteasome subunits. Previous proteomic screening showed that there were a large number of binding proteins in eggshell matrix; the 20 S proteasome is the catalytic core of the 26 S proteasome complex that represents the major component of the adenosine triphosphate (ATP)/ubiquitin-dependent protein degradation pathway. This pathway regulates many processes in the cells, such as progress through the cell cycle, gene transcription, and metabolic pathway (34). PSMB2 has been found in the bone marrow of human and plays a role in regulating homologous recombination (HR) and DNA double-strand break (DSB) repair in human cells (35–38). Studies in the pigs showed that proteasome inhibitors blocked the exit of maturing pig oocytes from the myocardial infarction (MI) stage (39). PSMB2 gene transcription had a positive effect on the meiosis of bovine oocytes (34). The above results show that the PSMB2 gene had an effect on follicular development and protein homeostasis, but its effect on eggshell quality is not clear. The results of the GWAS for the ESS trait showed that four SNP markers were located in a 3.77 kb region on GGA13. SLIT3 is one of the three SLIT ligands identified in the central nervous system, expressed and involved in muscle development and bone formation (40), angiogenesis (41), and stem cell migration (42), which are widely expressed in various cell types during embryogenesis and are involved in basic aspects of limb development. Knockdown of individual SLIT genes or Slit1 and Slit2 in combination resulted in a shortening of the length of the humerus (43). Kim et al. (44) found that β-catenin knockdown completely blocked Slit3-stimulated osteoblast migration and proliferation. The SLIT/ROBO pathway is associated with the development of regrading ovarian follicles in hens through autocrine or paracrine mode. Overexpression of SLIT3 can significantly reduce mRNA and protein expression levels of follicle-stimulating hormone (FSHR), growth and differentiation factor 9 (GDF9), osteogenesis acute regulatory protein (STAR), and cytochrome P450 11A1 (CYP11A1) in GC. The proliferation of primordial granulosa cells was significantly reduced. These results suggest that SLIT3 may inhibit the proliferation, differentiation, and follicular selection of granulosa cells (45). Meanwhile, the SLIT-ROBO pathway is also believed to play an important role in human angiogenesis and vascular system function. Reproductive tissues, such as ovaries, endometrium, and placenta, are rich in blood vessels and have dynamic and tightly regulated angiogenesis and remodeling. Eggshells are formed in the womb, and the rich blood vessels facilitate material exchange. Calcium for eggshell formation is provided by feed and bone tissue. The laying process of laying hens is a process of structural bone destruction. We hypothesized that SLIT3 regulates eggshell strength by affecting the proliferation, differentiation, absorption of bone cells, and changing the calcium content in bone tissue. All the above pieces of evidence suggested SLIT3 should be a crucial and promising candidate gene relating to eggshell quality.

### Haugh Unit

Haugh unit is an important index to evaluate egg protein quality and reflect egg freshness. It is calculated according to EW and egg height. Liu et al. (6) performed a GWAS with 1,078 hens aged 70 and 80 weeks and found that the genomic region from 8.95 to 9.31 Mb (≈0.36 Mb) on GGA13 was significantly correlated with the HU of eggs. In the present study, the TTLL7 gene is the only candidate gene. TTLL7 gene is highly expressed in the nervous system and encodes for a protein that is a cytosolic enzyme involved in the post-translational modification of alpha-tubulin, this microtubule can transfer nutrients that are needed during egg formation. In previous studies, Zhang et al. (1) showed that the TTL gene was a candidate gene for EW through the GWAS test on Jinghai Yellow chickens. It can be concluded that the TTLL7 gene affects HU by affecting EW.

## Conclusions

In this study, we report GWAS analysis for six egg-laying traits and egg quality in Lingkun chickens. A total of 37 significant SNPs were identified that include GAL, CENPF, GPC2, PEMT, TFAP2D, and CPQ genes related to egg-laying and SLC5A7, NCALD, PSMB2, SLIT3, and TTLL7 genes related to egg quality. In addition, the identified region on GGA13 for ESS was a promising QTL that could be potentially applied in marker assistant selection in a breeding program to improve egg quality, which needs further study. Our results found some potential genes, which might be used to improve egg production rate and egg quality.

## Materials and Methods

### Animals and Data Collection

The population selected for the study is an eighth generation pure line of Lingkun chicken from Wenzhou Golden Land Development Co. Ltd. The eighth generation was formed by 84 unrelated male chickens, each of which mated with 12 female chickens. There were 266 female offsprings that were chosen randomly from the same batch of chickens. The chickens were reared in stair-step cages under consistent conditions. We recorded their egg production every day. Eggs were collected in three consecutive days when hens were 72 weeks old. The average for 3 d was taken as the phenotypic value of each trait for every hen. Individual data for egg quality traits that included EW, EST, ESS, and HU were measured by instrument (Robotmation Co., Ltd., EMT-5200; Robotmation Co., Ltd., EFG-0503; Karl Deutsch, 1061). All phenotypic values of traits in genotyped individuals were tested for normality, and some abnormal values that were extremely deviating from normal distribution were deleted before conducting association tests.

### Sample Preparation

Blood samples were collected from the wing vein of 266 female offspring using heparin sodium as anticoagulants. The genomic DNA of each individual was extracted from Lingkun chicken using the E.Z.N.A. Tissue DNA Kit (Omega Bio-Tek) following the Tissue DNA – Spin Protocol. After the DNA samples were delivered, a quality control test was carried out on the specimens, and the qualified DNA (>3 μg; concentration >30 ng/μl; OD260/OD280 = 1.80–2.00) was used to do further study. All the samples in this experiment were tested using genomic DNA gelose gel electrophoresis and a total of 266 have reached the qualified requirements. Electrophoresis point holes were required to be clean and pollution-free, the main band was clear, and there was no trailing.

### Whole-Genome Resequencing

We chose Illumina pair-end sequencing (PE150) to do the resequencing project. For Illumina pair-end sequencing, at least 1 μg of genomic DNA was used for sequencing library construction for each sample. Paired-end libraries with insert sizes of ≈450 bp were prepared following Illumina's standard genomic DNA library preparation procedure. Purified genomic DNA is sheared into smaller fragments with the desired size by Covaris, and T4 DNA polymerase was applied to generate blunt ends. After adding an “A” base to the 3' end of the blunt-phosphorylated DNA fragments, adapters are ligated to the ends of the DNA fragments. The desired fragments can be purified through gel-electrophoresis, then selectively enriched and amplified by PCR. The index tag could be introduced into the adapter at the PCR stage as appropriate followed by a library quality test. After being quantified by TBS380, paired-end libraries were sequenced by Shanghai Biozeron Biotechnology Co., Ltd. (Shanghai, China) with the Illumina HiSeq platform according to the manufacturer's standard protocols. In this experiment, clean reads (72788874.44) were generated and 99.18% of these were mapped to the chicken genome ftp://ftp.ensembl.org/pub/release-76/fasta/.

### Variant Discovery and Quality Control

A total of 1,509,826,877 SNPs were obtained. Quality control of the genotyping data was performed in PLINK (v1.9). During this process, markers were selected based on three conditions: The SNPs that exhibited minor allele frequencies (MAFs) lower than 5%, low minor allele frequency lower than 2%, and a Hardy–Weinberg equilibrium tests lower than 1.0 × 10^−8^ were rejected. Finally, 266 individuals and 9,327,106 SNPs were identified in the present study. The experiment fast structure (https://github.com/rajanil/fastStructure) was used to perform population structure analysis. It was based on the maximum likelihood method (MLM). In addition, a principal component analysis (PCA) for the independent 9,327,106 SNPs was performed in Genome-wide Complex Trait Analysis (GCTA) (version 1.24). In order to reduce the influence of population stratification, the first and second components of the PCA were used in the model.

### Statistical Analysis

The general linear regression model (GLM) in PLINK was used in this study. The model is as follows:


$$\begin{array}{l} \text{Y}=\text{Xb}+\text{g}+\varepsilon ;Var\left(y\right)=\text{V}=A\sigma_{\text{g}}^{2}+I\sigma_{\varepsilon }^{2} \end{array}$$

where Y is the vector of observations; X is a design matrix of covariates containing all other fixed effects; *b is a vector matrix of fixed effects that includes* population structure, g is a vector of SNP effects with $g\;\sim \;N\;\left(0,A\sigma_{g}^{2}\right)$, A is interpreted as the genetic relationship matrix (GRM), which was calculated using the 9,327,106 independent SNPs acquired by PLINK (v1.9) software, ε is a vector of errors with $\varepsilon \;\sim \;N\;\left(0,I\sigma_{\varepsilon }^{2}\right)$. The analyses were performed using TASSEL v5.2 software (version 2.1). Significance thresholds were established from the estimated number of independent SNP markers and linkage disequilibrium (LD) blocks. LD block was defined as set D to 0.8 and the maximum interlocking area length to 500 KB. Using this approach, a total of 527,732 independent SNP markers and LD blocks were found. The threshold *p*-value of the 5% Bonferroni genome-wide significance was calculated based on the estimated number of independent SNPs and LD blocks for autosome SNPs. The threshold *p*-value of the 5% Bonferroni genome-wide significance was set at 9.47E-08 (0.05/527, 732), and the threshold *p*-value for significance was 1.89E-06 (1/527, 732).

The gene closest to the SNP with an extremely significant correlation is considered as a candidate gene. Sequence alignment was conducted through the National Center for Biotechnology Information (NCBI) and Ensembl databases to identify genes within 100 KB upstream and downstream of significant SNPs.

## Data Availability Statement

The datasets presented in this study can be found in online repositories. The names of the repository/repositories and accession number(s) can be found below: National Genomics Data Center, China National Center for Bioinformation (accession: https://bigd.big.ac.cn/gsa/browse/CRA006206).

## Ethics Statement

The experiment was approved by the Animal Care Committee of Anhui Agricultural University (No. SYDW-P20190600601).

## Author Contributions

LL, HC, SZ, YZ, SL, CW, YT, and TZ contributed to conception and design of the study. XL organized the database. WX performed the statistical analysis. JG wrote the first draft of the manuscript. All authors contributed to manuscript revision, read, and approved the submitted version.

## Funding

The authors are grateful for the financial support provided by Zhejiang Major Scientific and Technological Project on Agricultural (Livestock's) Breeding (2021C02068-10) and Wenzhou New Poultry Variety Breeding Cooperation Group Project (2019ZX005).

## Conflict of Interest

XL was employed by the company Wenzhou Golden Land Agricultural Development Co., Ltd. The remaining authors declare that the research was conducted in the absence of any commercial or financial relationships that could be construed as a potential conflict of interest.

## Publisher's Note

All claims expressed in this article are solely those of the authors and do not necessarily represent those of their affiliated organizations, or those of the publisher, the editors and the reviewers. Any product that may be evaluated in this article, or claim that may be made by its manufacturer, is not guaranteed or endorsed by the publisher.

## References

[1] Zhang GX Fan QC Wang JY Zhang T Xue Q Shi HQ. Genome-wide association study on reproductive traits in Jinghai Yellow Chicken. Anim Reprod Sci. (2015) 163:30–4. 10.1016/j.anireprosci.2015.09.011 26498507

[2] Liu Z Yang N Yan Y Li G Liu A Wu G. Genome-wide association analysis of egg production performance in chickens across the whole laying period. BMC Genet. (2019) 20:67. 10.1186/s12863-019-0771-7 31412760 PMC6693279

[3] Yuan J Sun C Dou T Yi G Qu L Qu L. Identification of promising mutants associated with egg production traits revealed by Genome-Wide association study. PLoS ONE. (2015) 10:e140615. 10.1371/journal.pone.0140615 26496084 PMC4619706

[4] Liu Z Sun C Yan Y Li G Wu G Liu A. Genome-wide association analysis of age-dependent egg weights in chickens. Front Genet. (2018) 9:128. 10.3389/fgene.2018.00128 29755503 PMC5932955

[5] Sun C Qu L Yi G Yuan J Duan Z Shen M. Genome-wide association study revealed a promising region and candidate genes for eggshell quality in an F2 resource population. BMC Genomics. (2015) 16:565. 10.1186/s12864-015-1795-7 26228268 PMC4521446

[6] Liu Z Sun C Yan Y Li G Shi F Wu G. Genetic variations for egg quality of chickens at late laying period revealed by genome-wide association study. Sci Rep. (2018) 8:10832. 10.1038/s41598-018-29162-7 30018363 PMC6050282

[7] Wang C Li SJ Yu WH Xin QW Li C Feng YP. Cloning and expression profiling of the VLDLR gene associated with egg performance in duck (Anas platyrhynchos). Genet Sel Evol. (2011) 43:29. 10.1186/1297-9686-43-29 21819592 PMC3162882

[8] Bai DP Hu YQ Li YB Huang ZB Li A. Polymorphisms of the prolactin gene and their association with egg production traits in two Chinese domestic ducks. Br Poult Sci. (2019) 60:125–9. 10.1080/00071668.2019.1567909 30648884

[9] Xie L Luo C Zhang C Zhang R Tang J Nie Q. Genome-wide association study identified a narrow chromosome 1 region associated with chicken growth traits. PLoS ONE. (2012) 7:e30910. 10.1371/journal.pone.0030910 22359555 PMC3281030

[10] Jin CF Chen YJ Yang ZQ Shi K Chen CK. A genome-wide association study of growth trait-related single nucleotide polymorphisms in Chinese Yancheng chickens. Genet Mol Res. (2015) 14:15783–92. 10.4238/2015.December.1.30 26634546

[11] Fernandez ME Goszczynski DE Liron JP Villegas-Castagnasso EE Carino MH Ripoli MV. Comparison of the effectiveness of microsatellites and SNP panels for genetic identification, traceability and assessment of parentage in an inbred Angus herd. Genet Mol Biol. (2013) 36:185–91. 10.1590/S1415-47572013000200008 23885200 PMC3715284

[12] Liao H Winkfein RJ Mack G Rattner JB Yen TJ. CENP-F is a protein of the nuclear matrix that assembles onto kinetochores at late G2 and is rapidly degraded after mitosis. J Cell Biol. (1995) 130:507–18. 10.1083/jcb.130.3.507 7542657 PMC2120529

[13] Hussein D Taylor SS. Farnesylation of Cenp-F is required for G2/M progression and degradation after mitosis. J Cell Sci. (2002) 115:3403–14. 10.1242/jcs.115.17.3403 12154071

[14] Yang ZY Guo J Li N Qian M Wang SN Zhu XL. Mitosin/CENP-F is a conserved kinetochore protein subjected to cytoplasmic dynein-mediated poleward transport. Cell Res. (2003) 13:275–83. 10.1038/sj.cr.7290172 12974617

[15] Crespo NC Ohkanda J Yen TJ Hamilton AD Sebti SM. The farnesyltransferase inhibitor, FTI-2153, blocks bipolar spindle formation and chromosome alignment and causes prometaphase accumulation during mitosis of human lung cancer cells. J Biol Chem. (2001) 276:16161–7. 10.1074/jbc.M006213200 11154688

[16] Filmus J Capurro M Rast J. Glypicans. Genome Biol. (2008) 9:224. 10.1186/gb-2008-9-5-224 18505598 PMC2441458

[17] Matas-Rico E van Veen M Leyton-Puig D van den Berg J Koster J Kedziora KM. Glycerophosphodiesterase GDE2 promotes neuroblastoma differentiation through glypican release and is a marker of clinical outcome. Cancer Cell. (2016) 30:548–62. 10.1016/j.ccell.2016.08.016 27693046

[18] Jacobs RL Zhao Y Koonen DP Sletten T Su B Lingrell S. Impaired de novo choline synthesis explains why phosphatidylethanolamine N-methyltransferase-deficient mice are protected from diet-induced obesity. J Biol Chem. (2010) 285:22403–13. 10.1074/jbc.M110.108514 20452975 PMC2903412

[19] Serafim V Chirita-Emandi A Andreescu N Tiugan DA Tutac P Paul C. Single nucleotide polymorphisms in PEMT and MTHFR genes are associated with omega 3 and 6 fatty acid levels in the red blood cells of children with obesity. Nutrients. (2019) 11:2600. 10.3390/nu11112600 31671528 PMC6893426

[20] Schreiweis MA Hester PY Settar P Moody DE. Identification of quantitative trait loci associated with egg quality, egg production, and body weight in an F2 resource population of chickens. Anim Genet. (2006) 37:106–12. 10.1111/j.1365-2052.2005.01394.x 16573524

[21] Goraga ZS Nassar MK Brockmann GA. Quantitative trait loci segregating in crosses between New Hampshire and White Leghorn chicken lines: I. Egg production traits. Anim Genet. (2012) 43:183–9. 10.1111/j.1365-2052.2011.02233.x 22404354

[22] Liao R Zhang X Chen Q Wang Z Wang Q Yang C. Genome-wide association study reveals novel variants for growth and egg traits in Dongxiang blue-shelled and White Leghorn chickens. Anim Genet. (2016) 47:588–96. 10.1111/age.12456 27166871

[23] Li X Glubrecht DD Mita R Godbout R. Expression of AP-2delta in the developing chick retina. Dev Dyn. (2008) 237:3210–21. 10.1002/dvdy.21744 18924234

[24] Girard M Bizet AA Lachaux A Gonzales E Filhol E Collardeau-Frachon S. DCDC2 mutations cause neonatal sclerosing cholangitis. Hum Mutat. (2016) 37:1025–9. 10.1002/humu.23031 27319779

[25] Grati M Chakchouk I Ma Q Bensaid M Desmidt A Turki N. A missense mutation in DCDC2 causes human recessive deafness DFNB66, likely by interfering with sensory hair cell and supporting cell cilia length regulation. Hum Mol Genet. (2015) 24:2482–91. 10.1093/hmg/ddv009 25601850 PMC4383862

[26] Gingras R Richard C El-Alfy M Morales CR Potier M Pshezhetsky AV. Purification, cDNA cloning, and expression of a new human blood plasma glutamate carboxypeptidase homologous to N-acetyl-aspartyl-alpha-glutamate carboxypeptidase/prostate-specific membrane antigen. J Biol Chem. (1999) 274:11742–50. 10.1074/jbc.274.17.11742 10206990

[27] Dunn AD Myers HE Dunn JT. The combined action of two thyroidal proteases releases T4 from the dominant hormone-forming site of thyroglobulin. Endocrinology. (1996) 137:3279–85. 10.1210/endo.137.8.8754751 8754751

[28] Suban D Zajc T Renko M Turk B Turk V Dolenc I. Cathepsin C and plasma glutamate carboxypeptidase secreted from Fischer rat thyroid cells liberate thyroxin from the N-terminus of thyroglobulin. Biochimie. (2012) 94:719–26. 10.1016/j.biochi.2011.10.018 22127294

[29] Sato T Abe T Chida D Nakamoto N Hori N Kokabu S. Functional role of acetylcholine and the expression of cholinergic receptors and components in osteoblasts. FEBS Lett. (2010) 584:817–24. 10.1016/j.febslet.2010.01.001 20067796

[30] Ma WW Ding BJ Yuan LH Zhao L Yu HL Xi YD. Neurocalcin-delta: A potential memory-related factor in hippocampus of obese rats induced by high-fat diet. Afr Health Sci. (2017) 17:1211–21. 10.4314/ahs.v17i4.32 29937895 PMC5870285

[31] Rudkowska I Perusse L Bellis C Blangero J Despres JP Bouchard C. Interaction between common genetic variants and total fat intake on low-density lipoprotein peak particle diameter: a Genome-Wide Association Study. J Nutrigenet Nutrigenomics. (2015) 8:44–53. 10.1159/000431151 26112879

[32] Mourot M Dufort I Gravel C Algriany O Dieleman S Sirard MA. The influence of follicle size, FSH-enriched maturation medium, and early cleavage on bovine oocyte maternal mRNA levels. Mol Reprod Dev. (2006) 73:1367–79. 10.1002/mrd.20585 16894554

[33] Sun C Xu G Yang N. Differential label-free quantitative proteomic analysis of avian eggshell matrix and uterine fluid proteins associated with eggshell mechanical property. Proteomics. (2013) 13:3523–36. 10.1002/pmic.201300286 24151251

[34] Bard J Goodall EA Greene ER Jonsson E Dong KC Martin A. Structure and function of the 26S proteasome. Annu Rev Biochem. (2018) 87:697–724. 10.1146/annurev-biochem-062917-011931 29652515 PMC6422034

[35] Gudmundsdottir K Lord CJ Ashworth A. The proteasome is involved in determining differential utilization of double-strand break repair pathways. Oncogene. (2007) 26:7601–6. 10.1038/sj.onc.1210579 17563742

[36] Collavoli A Comelli L Cervelli T Galli A. The over-expression of the beta2 catalytic subunit of the proteasome decreases homologous recombination and impairs DNA double-strand break repair in human cells. J Biomed Biotechnol. (2011) 2011:757960. 10.1155/2011/757960 21660142 PMC3110333

[37] Fagerberg L Hallstrom BM Oksvold P Kampf C Djureinovic D Odeberg J. Analysis of the human tissue-specific expression by genome-wide integration of transcriptomics and antibody-based proteomics. Mol Cell Proteomics. (2014) 13:397–406. 10.1074/mcp.M113.035600 24309898 PMC3916642

[38] Jacquemont C Taniguchi T. Proteasome function is required for DNA damage response and fanconi anemia pathway activation. Cancer Res. (2007) 67:7395–405. 10.1158/0008-5472.CAN-07-1015 17671210

[39] Chmelikova E Sedmikova M Rajmon R Petr J Svestkova D Jilek F. Effect of proteasome inhibitor MG132 on *in vitro* maturation of pig oocytes. Zygote. (2004) 12:157–62. 10.1017/S0967199404002734 15460111

[40] Holmes G Niswander L. Expression of slit-2 and slit-3 during chick development. Dev Dyn. (2001) 222:301–7. 10.1002/dvdy.1182 11668607

[41] Zhang B Dietrich UM Geng JG Bicknell R Esko JD Wang L. Repulsive axon guidance molecule Slit3 is a novel angiogenic factor. Blood. (2009) 114:4300–9. 10.1182/blood-2008-12-193326 19741192 PMC2774558

[42] Geutskens SB Andrews WD van Stalborch AM Brussen K Holtrop-de HS Parnavelas JG. Control of human hematopoietic stem/progenitor cell migration by the extracellular matrix protein Slit3. Lab Invest. (2012) 92:1129–39. 10.1038/labinvest.2012.81 22614124

[43] Rafipay A Dun XP Parkinson DB Erskine L Vargesson N. Knockdown of slit signaling during limb development leads to a reduction in humerus length. Dev Dyn. (2021) 250:1340–57. 10.1002/dvdy.284 33347679

[44] Kim BJ Lee YS Lee SY Baek WY Choi YJ Moon SA. Osteoclast-secreted SLIT3 coordinates bone resorption and formation. J Clin Invest. (2018) 128:1429–41. 10.1172/JCI91086 29504949 PMC5873876

[45] Xu R Qin N Xu X Sun X Chen X Zhao J. Implication of SLIT3-ROBO1/ROBO2 in granulosa cell proliferation, differentiation and follicle selection in the prehierarchical follicles of hen ovary. Cell Biol Int. (2018) 42:1643–57. 10.1002/cbin.11063 30288875

